# Maternal and Umbilical Cord Blood Levels of Lead and Cadmium in Sudanese Women with Preeclampsia

**DOI:** 10.3390/jcm15166307

**Published:** 2026-08-14

**Authors:** Alaeldin Elhadi, Manal N. Sharif, Hamdan Z. Hamdan, Ishag Adam, Mohamed F. Lutfi

**Affiliations:** 1 Sudan Academy of Sciences, Khartoum 11111, Sudan; 2 College of Medicine, King Khalid University, Abha 62521, Saudi Arabia; 3 Department of Pathology, College of Medicine, Qassim University, Buraydah 52571, Saudi Arabia; 4 Department of Obstetrics and Gynecology, College of Medicine, Qassim University, Buraydah 52571, Saudi Arabia; 5 Department of Physiology, College of Medicine, Qassim University, Buraydah 52571, Saudi Arabia; mf.lutfi@qu.edu.sa

**Keywords:** cadmium, lead, preeclampsia, pregnancy, age, umbilical cord, Sudan

## Abstract

**Background/Objectives:** Previous studies hypothesized that significant exposure to lead (Pb) and/or cadmium (Cd) induces preeclampsia in pregnant women with subsequent unfavorable obstetric outcomes. This study aimed to compare maternal (Pb and Cd) and umbilical cord (Pb and Cd) levels between patients with preeclampsia and healthy control pregnant women and investigate the variables that are associated with preeclampsia. In addition, the possible influences of these heavy metals on birth weight were assessed. **Methods**: A case–control study involving 60 women in each group was conducted at the Maternity Hospital in Omdurman, Sudan, between January and June 2021, and included pregnant women residing in Omdurman city, Khartoum state, Sudan. A structured questionnaire was used to gather clinical and medical history. Maternal and cord blood concentrations of Pb and Cd were measured using an atomic absorption spectrophotometer. Univariate and multivariate binary logistic regression analyses were conducted to identify variables associated with preeclampsia. **Results**: The average maternal age in cases was 25.2 (6.1) years, and in the control group, it was 27.7 (7.2) years. All included participants were residing in Omdurman city, Khartoum state. Preeclampsia patients exhibited significantly elevated mean (SD) levels of umbilical cord Pb [14.2 (5.3) vs. 10.2 (4.5) µg/L, *p* < 0.001], and median (25th–75th quartile) maternal Cd [3.5 (2.0–6.7) vs. 3.0 (2.0–4.0) µg/L, *p* = 0.021] and umbilical cord Cd [5.0 (3.0–8.1) vs. 2.5 (1.0–4.0) µg/L, *p* < 0.001] compared with healthy controls. Mean (SD) of umbilical cord Pb level was lower than maternal Pb [14.2 (5.3) vs. 15.7 (7.8) µg/L, *p* = 0.246), but not reached statistical significance, while median (25th–75th quartile) concentration of umbilical cord Cd and maternal Cd remained comparable [5.0 (3.0–8.1) vs. 3.5 (2.0–6.7) µg/L, *p* = 0.093], in patients with preeclampsia. Levels of umbilical cord Cd correlated inversely with birth weight [Sperman’s correlation coefficient (*rho*) = −0.689, *p* = 0.013]. Umbilical cord Pb [aOR = 1.29; 1.09 to 1.54; *p* = 0.003], and Cd [aOR = 1.37; 1.08 to 1.73; *p* = 0.007], in addition to maternal Cd [aOR = 1.35; 1.06 to 1.72; *p* = 0.012], were among the variables that are significantly associated with preeclampsia in univariate and multivariate analysis. **Conclusions**: Umbilical cord Pb, maternal Cd, and umbilical cord Cd concentrations were elevated in pre-eclamptic patients compared with healthy parturient controls and associated with preeclampsia. The preferential higher availability of Cd, but not Pb, in fetal compared with maternal blood might explain why umbilical cord Cd, but not umbilical cord Pb, had a significant negative effect on birthweight.

## 1. Introduction

Preeclampsia is a well-established primary driver of maternal mortality and morbidity during the second half of the pregnancy [1]. Hypertension, proteinuria, and/or end-organ dysfunction are the classic triad that characterizes patients with preeclampsia [2]. Studies on the pathogenesis of preeclampsia suggested several etiological mechanisms, including placental ischemia [3], endothelial dysfunction [4], infectious factors [5], genetic susceptibility [6,7], disturbed sympathovagal balance [8], deficiencies of essential trace elements [9,10], and heavy metal intoxication [11,12].

Lead (Pb) and cadmium (Cd) are among the heavy metals that can be transmitted across the placenta and induce detrimental effects on the fetus [13,14,15]. Alternatively, potential hypertensive effects of these heavy metals were demonstrated in exposed workers [16,17] and pregnant women [11,12]. Experimentally, hallmarks of preeclampsia were demonstrated following intraperitoneal injection of 0.5 mg/kg Cd in pregnant rats for two weeks [18]. Poor pregnancy outcomes were demonstrated in women with significantly increased levels of Pb and Cd, including low birth weight [19,20], decreased gestational age [21], and premature rupture of the membranes [22].

Both Pb and Cd leak into and contaminate agricultural soils [23], drinking water, rivers and sea fishes [24], and ambient air as a consequence of expanding industrial and anthropogenic activities. Major sources of environmental contamination include mining operations, the extensive use of phosphate fertilizers, fuel production and combustion, and the release of industrial emissions into the ecosystem [25]. In addition to cigarette smoking, improper disposal of batteries, electronic waste, and plastic materials further contributes to the environmental burden of these heavy metals [25].

It can be hypothesized that significant exposure to Pb and Cd can induce preeclampsia in pregnant women with subsequent preterm labor, decreased gestational age, and low birth weight. To further explore this hypothesis, we designed this study to compare maternal (Pb and Cd) and umbilical cord (Pb and Cd) levels of these heavy metals between pregnant women with preeclampsia and healthy controls. In addition, we assessed the effects of Pb and Cd levels on birth weight after matching the study groups for potential confounders, including maternal age [26], body mass index [27], childbearing history [28], and stage of pregnancy [29]. We expect the findings of the current study to significantly advance our understanding of the pathophysiology of preeclampsia among Sudanese women [30,31].

## 2. Materials and Methods

### 2.1. Study Settings and Populations

A case–control study was conducted in the labor ward of the Maternity Hospital, Omdurman, Sudan, from January to June 2021. Sixty parturient women who were diagnosed with preeclampsia were allocated to the case group, and an equal number of healthy pregnant women were allocated to the control group. All recruited participants resided in Omdurman, Khartoum State, Sudan. The strict definition of preeclampsia was followed: development of hypertension (systolic blood pressure ≥ 140 mm Hg or diastolic blood pressure ≥ 90 mm Hg) after 20 weeks of gestation in a previously normotensive woman, in addition to proteinuria (≥300 mg of protein in a 24 h urine sample or ≥2+ on dipstick) [2]. Preeclampsia was further divided into mild and severe based on diastolic blood pressure < 110 or ≥110 mmHg, respectively. Healthy parturient women in the same wards were allocated to the control group. Women with multiple pregnancies, stillbirth, endocrine disorders including thyroid disease and diabetes, essential hypertension, hepatic disease, and renal disease were excluded from both the case and control groups.

Subsequently, both groups signed a brief consent form; medical (including bad habits, e.g., smoking and alcohol consumption) and obstetric history (gravidity and gestational period) were gathered using a structured questionnaire. Maternal weight in kilograms and height in meters were documented, and body mass index (BMI) was computed via the equation weight (kg)/height (m)^2^. The newborn was weighed immediately, and his/her weight was recorded. Then, five mL of blood was collected from the maternal and umbilical cord vein immediately after delivery using a heparinized vacutainer.

### 2.2. Lead and Cadmium Quantification

The heparinized blood was allowed to freeze and kept at −20 °C until the analyses for Pb and Cd. A graphite furnace atomic absorption spectrophotometer (Thermo Electron, Cambridge, UK) was used to determine Pb [32] and Cd [33] levels in blood, as previously described. Very briefly, blood Pb concentrations were quantified using a calibration curve prepared from a 1000 µg/mL Pb standard solution diluted in 2% HNO_3_. All samples, calibrators, and quality-control reference materials were analyzed in duplicate following a 1:9 dilution. The method has a limit of detection of 0.1 µg/dL. Overall, precision and accuracy were 8.0% and 105.0%, respectively.

Cadmium was determined following preparation of the sample with Triton X-100 and nitric acid, followed by vortex mixing and centrifugation. The method demonstrated a precision of 7.0% and an accuracy of 19.0%, with a limit of detection (LOD) of 0.1 µg/L. Quality-control samples were included to ensure analytical performance.

### 2.3. Sample Size Calculation

The total sample size of 60 parturient women in both arms was computed assuming that this would have a significant difference in the mean outcome measures (Pb, Cd) and to have over 80% power to pick a difference of 5% at alpha  =  0.05. It has been assumed that 10% of women will either not cooperate or provide incomplete information.

### 2.4. Statistical Analysis

Data were entered into the Statistical Package for the Social Sciences (SPSS for Windows, version 16.0). The Shapiro–Wilk test was used to assess the distribution of the data. Normally distributed data were expressed as mean (SD), while non-normally distributed data were expressed as median (25th–75th interquartile). Student’s t-test and Mann–Whitney test were used to compare continuous data (Cd) between the preeclampsia and control groups. Binary logistic regression was conducted to identify the factors that may determine preeclampsia. Preeclampsia status was considered the dependent variable, and clinical characteristics and Pb and Cd levels were considered independent variables. Variables that reached a *p*-value ≤ 0.200 in univariate analysis were qualified to enter the multivariate regression analysis. Crude odds ratios (OR) and adjusted odds ratios (aOR) were reported along with their 95% confidence interval (95% CI). Spearman’s correlation between Pb and Cd levels and birth weight was calculated only for the case group. A probability value of less than 0.05 was determined as a cutoff to reject the null hypothesis.

## 3. Results

The two groups (60 women in each arm) were closely matched in maternal age, parity, gestational stage, BMI, and hemoglobin levels. Birth weight was significantly lower in the preeclamptic group; see Table 1. All the participants denied any history of smoking or alcohol consumption during or before the current pregnancy.

**Figure 1:**
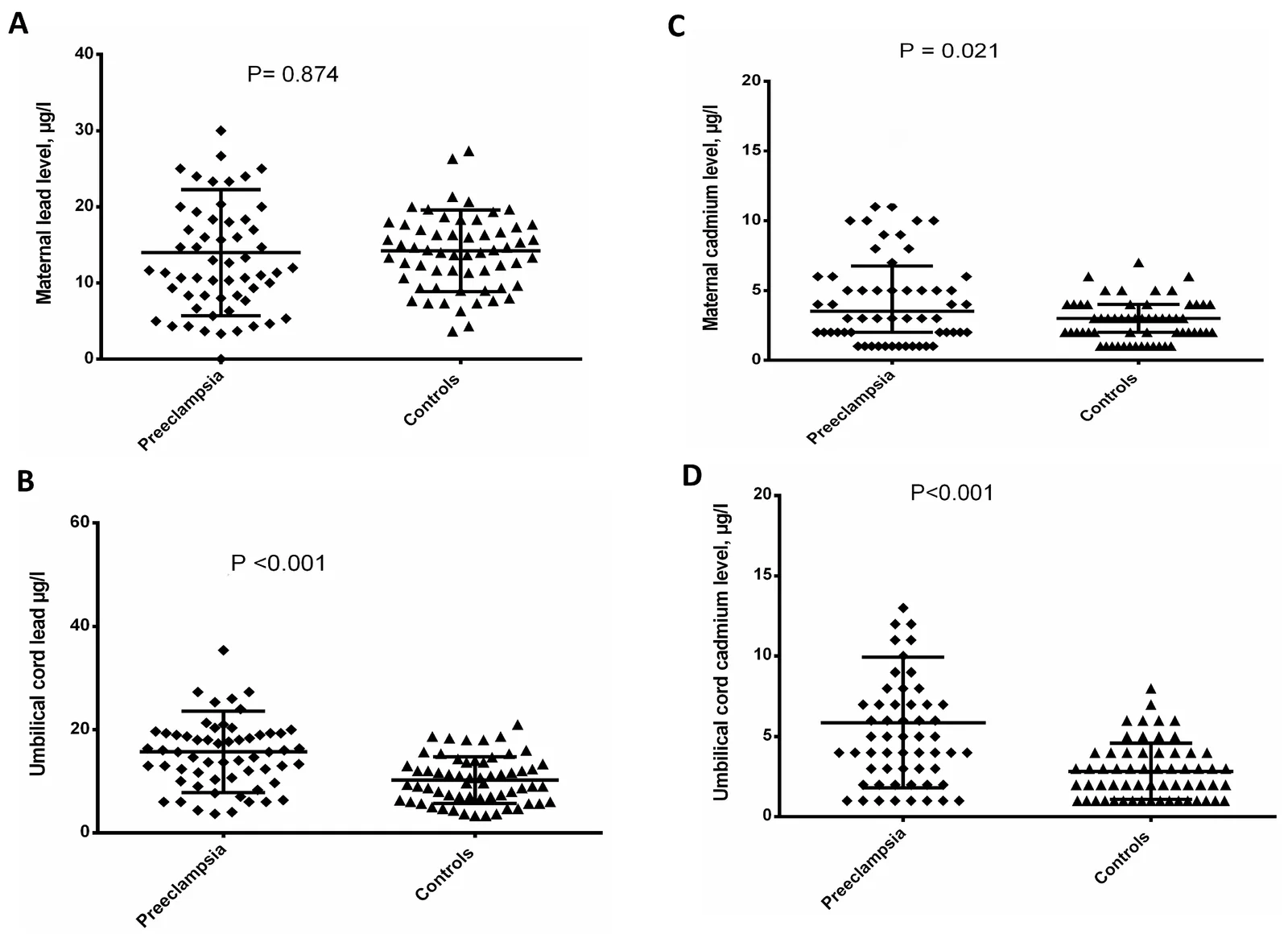
Boxplot figures show the comparison levels of lead and cadmium between cases and controls. (**A**) Compare the levels of lead between pregnant women with preeclampsia and controls. (**B**) Compare umbilical cord lead levels between pregnant women with preeclampsia and controls. (**C**) Compare the levels of cadmium between pregnant women with preeclampsia and controls. (**D**) Compare umbilical cord cadmium levels between pregnant women with preeclampsia and controls.

Patients with preeclampsia (53 and 7 women had severe and mild preeclampsia, respectively) achieved significantly higher mean (SD) levels of umbilical cord Pb [14.2 (5.3) vs. 10.2 (4.5) µg/L, *p* < 0.001], and median (25th–75th quartile) maternal Cd [3.5 (2.0–6.7) vs. 3.0 (2.0–4.0) µg/L, *p* = 0.021] and umbilical cord Cd [5.0 (3.0–8.1) vs. 2.5 (1.0–4.0) µg/L, *p* < 0.001] compared with healthy controls. Mean (SD) of umbilical cord Pb level was lower than maternal Pb [14.2 (5.3) vs. 15.7 (7.8) µg/L, *p* = 0.246], while the median (25th–75th quartile) concentration of umbilical cord Cd and maternal Cd remained comparable [5.0 (3.0–8.1) vs. 3.5 (2.0–6.7) µg/L, *p* = 0.093), in patients with preeclampsia, see Table 2 and Figure 1A–D.

Univariate binary logistic regression analysis showed that birth weight [OR = 0.99; 95% CI, 0.99 to 0.99; *p* < 0.001], gestational age [OR = 0.61; 95% CI, 0.43 to 0.86; *p* = 0.006], umbilical cord Pb [OR = 1.25; 95% CI, 1.12 to 1.39; *p* < 0.001], maternal Cd [OR = 1.21; 95% CI, 1.05 to 1.39; *p* = 0.006], and umbilical cord Cd [OR = 1.35; 95% CI, 1.17 to 1.56; *p* < 0.001] were associated with preeclampsia, see Table 3.

After running the multivariate regression model, all variables were found to be significantly associated with preeclampsia. That includes, hemoglobin levels [aOR = 2.81; 95% CI, 1.00 to 7.87; *p* = 0.048], parity [aOR = 0.56; 95% CI, 0.33 to 0.93; *p* = 0.026], birth weight [aOR = 0.99; 95% CI, 0.99 to 0.99; *p* < 0.001], gestational age [aOR = 0.47; 95% CI, 0.25 to 0.89; *p* = 0.020], umbilical cord Pb levels [aOR = 1.29; 95% CI, 1.09 to 1.54; *p* = 0.003], maternal Cd levels [OR = 1.35; 95% CI, 1.06 to 1.72; *p* = 0.012], and umbilical cord Cd [aOR = 1.37; 95% CI, 1.08 to 1.73; *p* = 0.007], see Table 3.

Although no significant correlation was found between Pb and Cd levels and maternal blood pressure, umbilical cord Cd correlated inversely with birth weight (rho coefficient = −0.689, *p* = 0.013), as shown in Table 4.

## 4. Discussion

The present study was designed to test the hypothesis that exposure to environmental Pb and Cd is associated with preeclampsia and impaired fetal growth. Although the specific sources of heavy metal exposure in our study population were not investigated, environmental contamination of food, drinking water, ambient air, and other anthropogenic sources have been recognized as the principal route of human exposure in previous studies [24,25]. Following maternal exposure, Pb and Cd may accumulate in the maternal circulation and cross the placenta, thereby contributing to placental dysfunction and adverse pregnancy outcomes [13,14,15].

The current findings demonstrated higher umbilical cord Pb, maternal Cd, and umbilical cord Cd levels in preeclamptic patients than in healthy pregnant controls. This points to the potential insult of Pb and Cd as environmental pollutants in the first group. Comparable findings were reported in several previous studies [34,35]. In a recent Egyptian study, Pb and Cd levels were significantly higher in 80 preeclamptic patients compared with 50 apparently healthy women, regardless of age, residence, education, smoking, and parity [35]. The same study demonstrated higher-than-normal levels of Cd in mothers with a previous history of chronic hypertension as well as those with a family history of preeclampsia. Likewise, Wang and colleagues reported similar findings [36]. Following evaluation of maternal Cd, umbilical cord Cd, and Cd levels in the amniotic fluid of 37 normotensive and 23 hypertensive women during the last trimester of pregnancy, Kosanovic et al. identified high Cd levels as a key factor in the development of hypertension among pregnant women smokers [37]. Experimentally, Wang and colleagues demonstrated hallmarks of preeclampsia in rats injected intraperitoneally with 0.5 mg/kg Cd from day 5 to day 19 of pregnancy [18]. A very recent study linked Cd accumulation in the placenta to the induction of FOXO3a, which contributes to trophoblast apoptosis and precipitates placental ischemia and preeclampsia [38]. Another mechanism proposed by Li and co-workers is that elevated maternal and placental Cd levels downregulate thyroid hormone receptor α expression in the placenta, thereby interfering with the angiogenic action of thyroid hormone [39]. Moreover, the same study reported that elevated Cd levels induce sFLT expression and, in turn, suppress PLGF and VEGF expression.

Regionally, in a Saudi study, Jameil compared maternal Pb levels of healthy normotensive pregnant ladies (Control group) with women at high risk of preeclampsia (HR group) and patients with preeclampsia (PE group) [40]. The results demonstrated a significantly increased maternal Pb level in the PE group compared with the controls. Although the maternal Pb level in the HR group was higher than in the controls and lower than in the PE group, these differences did not reach statistical significance.

The present study did not demonstrate a significant correlation between maternal Pb and maternal blood pressure. In a comparable study assessing the incidence of preeclampsia following environmental exposures of Iranian women to heavy metals, one unit increase in the common logarithm of umbilical cord Pb increased the risk of preeclampsia 12.96 times in patients with no occupational Pb exposure [34]. Interestingly, the umbilical cord Pb level was a more sensitive indicator of maternal reproductive toxicity compared with maternal Pb. This implication is further supported by our present results, which demonstrated higher umbilical cord Pb levels in preeclamptic patients than in healthy control women, whereas maternal Pb levels were comparable between the two groups.

The present results demonstrated that umbilical cord Cd levels are inversely related to newborn birth weight. Matching on maternal age [26], body mass index [27], childbearing [41], parity [28], and gestational age [29] between the groups currently studied precludes these factors as potential confounders of the negative influence of Cd on birth weight. This conclusion contradicts the implication of Nishijo et al., who attributed the lower birth weight of mothers with higher urinary Cd to an increased rate of preterm deliveries among them [42]. Alternatively, it was hypothesized that low birth weight associated with prenatal exposure to Cd is secondary to the simultaneous increase in glucocorticoids during intrauterine life [18,43]. Oral administration of Cd to experimental pregnant rats was associated with low birth weight deliveries but higher plasma corticosterone levels in mothers as well as newborns [43]. Although high maternal glucocorticoids explained most of the features of preeclampsia and low birth weight associated with Cd exposure, the cause of this hormonal derangement remained mysterious. Moreover, Wang et al. demonstrated, for the first time, that the placenta is an extra-adrenal source of high glucocorticoid levels associated with Cd-induced preeclampsia [18]. According to Wang et al.’s data, the expression of enzymes needed for corticosteroid synthesis was enhanced, while the principal negative regulator of placental glucocorticoids was suppressed following exposure to Cd.

On the other hand, the association between maternal Pb and low birth weight was documented in several previous reports; however, this inverse relationship did not reach statistical significance in the present results. Increased concentrations of Pb were observed in placentas from mothers who delivered low-birth-weight neonates [44]. Based on maternal Pb readings of 43,288 women enrolled in the New York State Heavy Metals Registry and the birth weight of their babies, a 5 and 10 μg/dL increase in maternal Pb was associated with about 61 and 87 g decreases in birth weight, respectively. Interestingly, a fivefold increase in the odds of low birth weight was reported in infants whose fathers suffered from occupational Pb exposure [45]. Low birth weight deliveries of Pb-exposed pregnant women were repeatedly attributed to reduced gestational age [46], probably due to premature rupture of the membranes [47]. This may explain the lack of a significant association between birth weight and Pb levels in our studied groups, which were matched for gestational age. Notably, umbilical cord Pb levels were lower than maternal Pb levels, whereas umbilical cord Cd and maternal Cd remained comparable among patients with preeclampsia we studied. This preferential higher availability of Cd in fetal blood might be another explanation for the significant inverse correlation between birth weight and umbilical cord Cd levels, but not umbilical cord Pb levels. However, the lower umbilical cord Pb measurements relative to maternal Pb remain to be explained by further research.

The current findings also highlight the importance of minimizing maternal exposure to heavy metals before and during pregnancy. However, the major sources of Pb and Cd exposure in our study population remain uncertain. Yet, other studies identified the environmental, dietary, and occupational exposure pathways as potential sources [48,49]. In regions at risk of environmental contamination such as our setting, strengthening environmental monitoring, improving industrial waste management, and incorporating exposure risk assessment into routine antenatal care may help reduce maternal and fetal exposure to toxic metals [49]. From a prophylaxis perspective, adequate maternal intake of essential micronutrients, particularly iron and calcium, may reduce the gastrointestinal absorption of Pb and Cd and potentially mitigate their adverse effects during pregnancy [14,39].

Another finding in this study is that multivariable logistic regression analysis demonstrated that umbilical cord blood Pb, umbilical cord blood Cd, and maternal Cd concentrations were independently associated with preeclampsia after adjustment for potential confounders. Each unit increase in umbilical cord Pb, umbilical cord Cd, and maternal Cd was associated with a 29%, 37%, and 35% increase in the odds of preeclampsia, respectively. These suggest that maternal and fetal exposure to Pb and Cd, together with maternal Cd burden, may contribute to the pathogenesis of preeclampsia independently of conventional obstetric risk factors. The stronger association observed for umbilical cord metals further supports the hypothesis that placental transfer and fetal accumulation of toxic metals may reflect placental dysfunction, oxidative stress, endothelial injury, and impaired angiogenesis, all of which are recognized mechanisms underlying preeclampsia [49,50,51]. Similar independent associations between elevated maternal or cord blood Pb and Cd concentrations and increased risk of preeclampsia have been reported in previous epidemiological studies and systematic reviews [36,52,53].

As per the existing literature, this study is the first to assess Pb and Cd levels in a patient with preeclampsia in Sudan. However, when interpreting the results of this study, some limitations should be considered. First, although all participants in both groups denied any history of active or passive smoking, we did not inquire about the husband’s occupation as a potential source of heavy metal exposure. Second, we did not investigate environmental and socioeconomic factors, including dietary habits, occupational and para-occupational exposures, residential environmental pollution, and household contamination, which may have influenced the observed associations between heavy metal concentrations and preeclampsia. Third, because this was a case–control study, the temporal relationship between heavy metal exposure and the development of preeclampsia could not be established. Consequently, the observed associations should not be interpreted as evidence of causality. Therefore, further study with a longitudinal design is needed.

## 5. Conclusions

In this setting, umbilical cord Pb, umbilical cord Cd, and maternal Cd were associated with preeclampsia. The present study demonstrated significantly higher umbilical cord blood Pb, maternal Cd, and umbilical cord Cd concentrations in women with preeclampsia than in healthy pregnant controls, supporting our hypothesis that increased maternal exposure to these heavy metals may contribute to the pathophysiology of preeclampsia and adversely affect fetal growth. Although umbilical cord Pb concentrations were lower than maternal Pb levels, umbilical cord and maternal Cd concentrations were comparable, suggesting greater fetal availability of Cd than Pb. This differential transplacental transfer may explain the significant inverse association between umbilical cord Cd, but not Pb, and birth weight. Collectively, these findings provide further evidence supporting the potential involvement of Pb and Cd in adverse pregnancy outcomes and emphasize the importance of reducing maternal exposure to these toxic metals during pregnancy. Future prospective longitudinal studies are needed to identify the major sources of exposure, clarify the temporal relationship between heavy metal exposure and preeclampsia, and determine whether targeted preventive interventions can improve maternal and neonatal outcomes.

## Figures and Tables

**Table 1:** Sociodemographic and clinical variables of parturients with preeclampsia and controls.

Variable	Preeclampsia (Number = 60)	Controls (Number = 60)	*p*-Value
Hemoglobin, g/dL	10.1(1.1)	10.0(0.9)	0.801
Maternal age, years	25.2 (6.1)	27.7 (7.2)	0.081
Gestational age, weeks	39.3 (1.2)	39.8 (1.2)	0.436
Parity, numbers	1.4 (1.5)	2.2 (2.3)	0.073
Birth weight, g	2615.7 (487)	3125.4 (398)	<0.001
BMI, kg/m^2^	24.2 (2.9)	23.9 (2.4)	0.526

**Table 2:** Mean (SD) or median interquartile of maternal and umbilical cord levels of lead and cadmium in the preeclamptic cases and controls.

Heavy Metal	Preeclampsia (Number = 60)	Control (Number = 60)	*p*-Value
Maternal Pb, µg/L	15.7 (7.8)	13.9 (8.3)	0.874
Umbilical cord Pb, µg/L	14.2 (5.3)	10.2 (4.5)	<0.001
*p*-value	0.246	<0.001	
Maternal Cd, µg/L	3.5 (2.0–6.7)	3.0 (2.0–4.0)	0.021
Umbilical cord Cd, µg/L	5.0 (3.0–8.1)	2.5 (1.0–4.0)	<0.001
*p*-value	0.093	0.727	

**Table 3:** Univariate and multivariate binary regression analysis for the variables associated with preeclampsia.

Variables	Univariate Analysis		Multivariate Analysis	
	OR (95% CI)	*p*-Value	aOR (95% CI)	*p*-Value
Hemoglobin	1.29 (0.88–1.90)	0.186	2.81(1.00–7.87)	0.048
Maternal age	0.97(0.92–1.03)	0.393		
Parity	0.79(0.62–1.00)	0.055	0.56(0.33–0.93)	0.026
Birth weight	0.99 (0.99–0.99)	<0.001	0.99 (0.99–0.99)	<0.001
BMI	1.04(0.91–1.19)	0.523		
Gestational age	0.61(0.43–0.86)	0.006	0.47 (0.25–0.89)	0.020
Maternal Pb	0.97(0.93–1.02)	0.345		
Umbilical cord Pb	1.25(1.12–1.39)	<0.001	1.29(1.09–1.54)	0.003
Maternal Cd	1.21(1.05–1.39)	0.006	1.35 (1.06–1.72)	0.012
Umbilical cord Cd	1.35 (1.17–1.56)	<0.001	1.37(1.08–1.73)	0.007

**Table 4:** Correlation between maternal and umbilical cord levels of lead and cadmium.

Variables	Maternal Pb		Maternal Cd		Umbilical Cord Pb		Umbilical Cord Cd	
	rho	*p*	rho	*p*	rho	*p*	rho	*p*
Birth weight	−0.438	0.628	−0.072	0.500	−0.352	0.378	−0.689	0.013 *
Maternal Pb	—	—	0.025	1.000	0.001	0.995	0.486	0.356
Maternal Cd	0.025	1.000	—	—	−0.025	1.000	−0.658	0.242
Umbilical cord Pb	0.001	0.995	−0.025	1.000	—	—	−0.257	0.658
Blood Pressure	−0.019	0.886	0.073	0.582	0.137	0.297	0.184	0.159

* *p*-value < 0.05.

## Data Availability

The data presented in this study are available on request from the corresponding author due to privacy concerns.
